# The study on the relationship between venture capital, tolerance to failure, and enterprise innovation performance

**DOI:** 10.3389/fpsyg.2023.1133324

**Published:** 2023-02-15

**Authors:** Jin Han, Minling Chen, YanXin Chen, Qinling Jing, ShuangYan Wang

**Affiliations:** ^1^School of Business, Xi’an University of Finance and Economics, Xi’an, China; ^2^School of Economics and Management, Xi’an ShiYou University, Xi’an, China; ^3^Foreign Languages School, Kunming University, Kunming, China; ^4^School of Economils and Management, Kunming University, Kunming, China

**Keywords:** venture capital, tolerance to failure, innovation performance, joint investment, geographical proximity

## Abstract

Venture capital not only affects enterprise innovation decisions by providing funds, value-added services and allocating control rights, but also the psychological capital of venture capital can enhance its tolerance for failure in innovation activities of enterprises, and thus have a positive impact on innovation performance of enterprises. This paper uses multivariate and negative binomial regression models, propensity score matching method and Heckman treatment effect model to study the impact mechanism of venture capital on enterprise innovation performance, and the mediation role of venture capital’s tolerance for innovation failure in the relationship between the above two; this paper studies the moderating effect of the characteristics of heterogeneous venture capital institutions, such as joint investment strategies and geographical proximity, on the relationship between venture capital failure tolerance and enterprise innovation performance. The results show that venture capital can significantly improve its tolerance for enterprise innovation failure by holding shares and occupying seats on the board of directors of enterprises, thereby bring the increase of the innovation performance of enterprises; if joint investment strategy and close investment are selected, the tolerance of venture capital to innovation failure will have a more obvious effect on the promotion of enterprise innovation performance.

## 1. Introduction

At present, China’s economy is developing in the direction of high quality, and innovation is an important basis for promoting high-quality economic development. The report of the 20th CPC National Congress states that the implementation of the innovation-driven development strategy should be accelerated, the main role of enterprises in scientific and technological innovation should be strengthened, and the level of transformation and industrialization of scientific and technological achievements should be improved. In this context, innovative enterprises are important carriers of technological innovation, and the effectiveness of the implementation of the national innovation-driven development strategy depends on the continuous improvement of the technological innovation capacity of these enterprises.

In the 2022 government work report, it is proposed that in the further implementation of the innovation-driven development strategy, it is required to promote the development of venture capital and improve the level of financial products and services in the field of innovative science and technology. Domestic and international empirical studies also suggest that venture capital, as an innovative way of financing, is well integrated with technology. In addition to providing companies with the capital necessary, venture capital institutions can also provide value-added services such as management supervision, which play an extremely important role in technological innovation and the transformation of technological achievements. As evidenced by the Venture Capital Development Report, at the end of 2018, the number and amount of venture capital invested in high-tech enterprises reached 9,279 and 175.72 billion RMB, respectively.

Enterprises need to invest a lot of capital in the process of innovation, which is characterized by a relatively long R&D cycle and high risks. The entry of venture capital can help companies to innovate, not only by addressing the lack of capital for research and development but also by providing value-added services in the areas of consulting and management supervision with its own advantages in industries functions and resources endowment, which can contribute to the improvement of enterprises’ innovation performance. However, due to the constraints of investment objectives and investment cycles, there is a risk of conflict between the short-term objectives of venture capital and time consuming in innovation activities of firms, which may probably lead to a suppression of corporate technological innovation brought by venture capital. Then will venture capital promote or inhibit business innovation?

As innovative and entrepreneurial enterprises are newly established and their assets can be highly earmarked, there is an information asymmetry between the investing parties, which can lead to mutual hedging. As a result, the configuration of corporate control becomes a common concern for entrepreneurs and venture capitalists to protect their respective interests. And organizational control theory, based on the economics of innovation, states that corporate control rights affects critical decisions regarding corporate innovation so that it further has a significant impact on corporate technological innovation (Xu and Xu, 2012). In the context of venture capital participation, further insight into the mechanisms by which venture capital affects the innovation performance of firms can be found in the control of the firm owned by the venture capitalist (ownership of equity and the right to sit on the board of directors of firms).

Corporate technological innovation is a long and risky process with a long R&D cycle, and the process of innovation requires risk-taking and tolerance from key players (He and Tian, 2020). Besides, the failure tolerance from venture capital institutions reflects their ability to accommodate corporate innovation failures and take risks, and attitudes towards technological innovation failure of a firm can influence corporate culture in terms of tolerance of innovation failures, as well as the attitude of entrepreneurs towards technological innovation failure, which can, in turn, have a significant impact on the development of technological innovation activities and the output of innovation outcomes (Tian and Wang, 2014). The question is therefore posed whether the tolerance of venture capitalists to innovation failure has an impact on their relationship between venture capital and enterprise innovation performance. This subject is important to corporate technological innovation--which is at the core of this paper’s research.

Research by domestic and international scholars on the impact of venture capital on corporate innovation performance has been conducted in two major areas:(1) Investigate the impact of venture capital participation on the technological innovation performance of enterprises. (2) The impact of the characteristics of venture capital institutions on technological innovation performance. However, existing research has neglected to examine in depth the mechanisms by which venture capital affects firms’ innovation performance. Moreover, considering that the tolerance of venture capital to technological innovation failure of a firm may affect the formation of a fault-tolerant culture and the willingness of entrepreneurs to innovate, it is necessary to investigate the impact of venture capital tolerance to innovation failure concerning the innovation performance of firms. In addition, there is a certain heterogeneity between different types of venture capital institutions, with significant differences in their joint investment strategies and geographical proximity. The relationship between venture capital firms’ tolerance for innovation failure and firm innovation performance is further affected by the differences in their joint investment strategies and geographical proximity.

Based on this, firstly, this paper studies the impact of venture capital participation on corporate innovation performance, and the mechanisms by which venture capital affects corporate innovation performance in terms of corporate control rights (ownership of corporate equity, ownership of corporate board seats). Secondly, the mediating effect of a venture capital’s tolerance for innovation failure between its shareholding, sending directors to occupy board seats, and the firm’s innovation performance is examined. Finally, the moderating effect of these characteristics of heterogeneous venture capital institutions on the relationship between their failure tolerance and firm innovation performance is investigated from two perspectives: joint investment strategy and geographical proximity. Our research provides a theoretical basis for an in-depth study of the relationship among venture capital participation, venture capital tolerance for innovation failure, and corporate innovation performance, as well as an empirical reference for building a fault-tolerant corporate culture and promoting corporate technological innovation.

## 2. Literature review

### 2.1. Research on the impact of venture capital participation on corporate innovation

In the research on the impact of venture capital participation on corporate innovation, domestic and foreign scholars have not yet reached a consistent conclusion.

On the one hand, some scholars have found that venture capital participation can reduce information asymmetry and moral hazard problems by providing value-added and supervision services, which can reduce the cost of external financing of enterprises, ultimately promote enterprise innovation. For example, Gu and Qian (2019) used data from Chinese listed companies and found that venture capital promotes enterprise innovation. Li and Yan (2020) found that venture capital can help enterprises make full use of resources spilled over from other firms, thus so as to have a positive impact on the innovation performance of enterprises. In addition, scholars have also found that by improving the internal and external governance mechanisms of firms and reducing uncertainty in the firm’s external environment, venture capital can increase the firm’s innovation output. For example, Feng et al. (2020) found that venture capital used their extensive management experience and resources to help improve the internal governance structure of firms, ultimately enhancing innovation performance. Zhang et al. (2021) found that venture capital can effectively improve firms’ innovation performance by reducing the uncertainty they face in the R&D process and increasing their risk tolerance. Dong et al. (2021)combined found that VC support not only improves firms’ success rate of patent application but also increases the innovation “value” represented by increased citation. Leogrande et al. (2021) found that the level of Venture Capitalist Expenditure is positively associated to Innovation Index.

On the other hand, due to the high risks faced by firms in the process of innovation, the long cycle of innovation returns, and limited by the objective of stage investment, venture capital eagered to recover their capital quickly, thus led to its potential adverse impact on firms’ technological innovation. For example, Arvanitis and Stucki (2014) found that firms supported by venture capital did not achieve the objective of improving innovation performance. Cheng and Zou (2020) found that after entering firms, venture capital did not promote higher levels of R&D investment or increased the number of patent applications. Xia and Le (2021) found that venture capital holding equity in firms harmed the level of innovation inputs and output results of firms. Leogrande et al. (2021)found that the level of Venture Capitalist Expenditure is negatively related to government procurement of advanced technology products, medium and hightech products exports.

### 2.2. Research on the impact of shareholding and dispatching directors of venture capital institutions on corporate innovation performance

Some academics have found that venture capital with shares and board seats can contribute to the innovation performance of companies by increasing their investment in innovation. For example, Gou and Dong (2014) found that the higher the percentage of corporate higher shareholding, the more the venture capital will induce the firm to invest more in innovation by taking control of enterprises. Celikyurt et al. (2014) found that after venture capital owned corporate board seats, firms’ R&D efforts, innovation output, and other transactional activities would be increased. However, some scholars have come to the opposite conclusion, for example, Wang and Zhou (2017) found that the shareholding ratio of the parent company of corporate venture capital can both promote and inhibit the R&D investment level, invention patents, and the total number of patent applications.

### 2.3. A study of the impact of venture capital characteristics on corporate innovation performance

Regarding the study of the attitudes of venture capital towards innovation failures affecting firm innovation, some scholars have found that different venture capital institutions will differ in their attitudes towards innovation failures in firms, which in turn will have different impacts on firm innovation. For example, Chemmanur et al. (2014) compare corporate venture capital with independent venture capital and find that corporate venture capital has a longer investment horizon, is more inclusive of failure, and is better at improving the innovation performance of firms. Tian and Wang (2014) found that the degree of tolerance of venture capital for innovation failure a firm can incentivize better innovation. Wang and Zhou (2017) found that firms that received corporate venture capital funding had significantly higher R&D investment than other firms, while for firms that received funding from independent venture capital, their patent applications were higher than those of other companies.

In terms of investment strategy, some scholars believe that joint investment provides help for enterprises’ technological innovation by providing complementary resources. For example, Chen et al. (2017) found that the association of multiple venture capital brings a stronger role in promoting enterprise innovation. Zhang (2020) found that under the joint investment strategy, the total number of enterprise patent applications and invention patent applications will be more. However, academics have also found that conflicts may arise between different investment institutions due to differences in strategies, objectives, and cultural differences, resulting in their inability to promote technological innovation in their firms. For example, Dong et al. (2019) found that the number of indirect linkages of competitors formed between venture capital institutions due to co-investment volume can hurt the innovation output of firms.

A review of the above-mentioned scholars’ research findings reveals that, firstly, although some scholars have focused on the impact of venture capital participation on firms’ innovation capabilities and performance, they have not conducted further in-depth studies on the mechanisms by which venture capital affects firms’ innovation performance. Secondly, although some scholars have paid attention to the fact that the attitude of venture capital to innovation failures in the early stage of enterprises may affect the risk appetite of entrepreneurs, they have not further studied the influence of venture capital institutions’ tolerance for innovation failures of enterprises on the relationship between venture capital and enterprise innovation performance. Finally, some studies have paid attention to the impact of the type, investment strategy of venture capital institutions on the innovation performance of enterprises, but due to the different characteristics of joint investment strategy and geographical proximity, some studies have ignored that different types of venture capital have different characteristics due to their own characteristics, and it will have a differential moderating effect on the relationship between the tolerance of innovation failure of enterprises and the innovation performance of enterprises.

By contrast, this paper contributes in several ways. Firstly, based on innovation economics and organizational control theory, the impact of venture capital participation on enterprise innovation performance is studied, and the mechanism of venture capital on enterprise innovation performance is studied from two aspects: shareholding ratio and sending directors to occupy seats on the corporate board of directors, it is of great significance to improve the internal governance mechanism and improve the innovation performance of enterprises. Secondly, based on the perspective of enterprise innovation culture, the mediating effects of the tolerance of venture capital to innovation failure between its shareholding ratio, the number of dispatched directors occupying the board seats and the innovation performance of enterprises are further investigated. Thirdly, the moderating effect of characteristics such as joint investment strategies and geographical proximity of investment institutions on the relationship between failure tolerance of venture capital and firm innovation performance is investigated from the perspective of the existence of heterogeneity of different types of investment institutions, it provides experience for improving the failure tolerance of venture capital and improving the innovation performance of enterprises. Fourthly, to better identify the “selection effect” and “value-added effect” of venture capital, this paper also adopts the PSM method and Heckman treatment effect model to further test the robustness of the shareholding ratio of venture capital, the impact of dispatched directors on the innovation performance of enterprises, and the mediating effect of failure tolerance of venture capital.

## 3. Theoretical analysis and research hypotheses

### 3.1. Effect of venture capital participation on the innovation performance of enterprises

Due to the relatively long cycle of various technological innovation activities carried out by enterprises, the risk of innovation failure is also very high, which makes enterprises often have problems of insufficient investment in R&D funds, etc., and venture capital can solve the problems faced by enterprises such as insufficient R&D investment funds by providing the funds needed by enterprises (Hsu et al., 2014).

If venture capital institutions are willing to invest in enterprises, the positive signals on enterprise operation and innovation will be transmitted to the capital market to certify the quality of enterprises, so that investors are willing to increase the funds invested in enterprises, and then enterprises will use the funds obtained from external investors for technology research and development, accelerating the process of enterprise innovation.

Since there will be a lot of uncertainty in the process of research and development, venture capital can rely on its own professional investment experience, technology and resources to provide enterprises with the professional knowledge and technical resources needed to carry out technological innovation, give strategic guidance to enterprises, and help enterprises innovate research and development models (Dong et al., 2017), reduce the cost of enterprise technological innovation (Lin and Zhang, 2019), thus increasing the efficiency of their technological innovation.

The spillover effect of R&D activities of enterprises often exists, and the resources and information of technological R&D spillover from the industry can realize the flow and dissemination of knowledge among enterprises, and enterprises can grasp the latest technology in the industry, which can avoid repetitive technological innovation and encourage enterprises to engage in innovation with higher technological content through the “demonstration effect” among enterprises. There is a general exchange of innovation resources between firms receiving funding from the same venture capital institutions (González-Uribe, 2020). Venture capital can not only provide funds for these enterprises with complementary innovation resources, but also provide them with useful decision-making support and technical guidance with their industry expertise, resource endowments, and social network in the process of carrying out innovation activities, and through the sharing of innovation resources, it is conducive to the overflow of innovation resources and core technologies obtained by enterprises in the same industry for their technological innovation. Therefore, the hypothesis is formulated:

*H1*: Venture capital participation can have a catalytic effect on the innovation performance of enterprises.

### 3.2. The mechanism of venture capital affecting the innovation performance of enterprises

Whereas organizational control theory based on the economics of innovation states that by influencing important decisions regarding firm innovation, the allocation of control over a firm ultimately affects its technological innovation performance (Xu and Xu, 2012). The control rights of an enterprise is mainly reflected in the voting rights (shareholding ratio) and the right to seat on the board of directors. The shareholding ratio of venture capital reflects the level of enterprise control rights and capital investment they have, and the board of directors is the most important decision-making and supervisory body in the corporate governance structure. After investing capital into enterprises, venture capital often try to occupy the seats of the board of directors of enterprises and grasp the decision-making power of important matters of enterprises, strengthen its position and role in the board of directors, improve its monitoring level of enterprises and the corporate governance structure. Therefore, this paper mainly uses the two variables of venture capital shareholding ratio and sending directors to occupy board seats of enterprises to measure the degree of control rights over enterprises, and on this basis, the influence mechanism of venture capital on the innovation performance of enterprises is studied from these two aspects.

#### 3.2.1. The impact of venture capital shareholding on the innovation performance of enterprises

The innovation activities of enterprises are often characterized by high risks, long R&D cycles, and information asymmetry, so when signing investment contracts with enterprises, to obtain more investment returns, venture capital institutions will implement supervision and control of enterprises by allocating control rights to them (Dong et al., 2017), improve the governance level of the enterprise (Xiong and Gui, 2018), influence important decisions in terms of corporate innovation, which in turn will have an impact on the activities carried out by the enterprise in terms of technological research and development and product development. The influence of venture capital shareholding on the innovation performance of companies is mainly reflected in the following aspects.

First of all, the shareholding ratio of venture capital reflects the level of capital invested and the control rights of the enterprise, the higher the shareholding ratio, the more capital and value-added services that venture capital can provide to enterprises, and can help enterprises with their own experience in project management and strategic guidance. In addition, it can reduce the R&D costs and expenses in the process of technological innovation of enterprises, and improve the innovation efficiency of enterprises by providing technical guidance and decision-making support to enterprises in technological innovation and product development.

Secondly, the shareholding ratio of venture capital institutions can send signals to the external capital market about the company’s operating conditions and innovation capabilities. The higher the shareholding ratio of investment institutions, the stronger the innovation ability and higher performance of enterprises, and the easier it is to attract funds from external investors, which can improve the willingness of entrepreneurs to innovate, which will increase the investment of enterprise R&D funds, avoid the failure of enterprises’ innovation projects due to financial constraints, and ultimately help realize the value-added of enterprise innovation value.

Finally, by their equity holdings in the enterprise, venture capitalists can strengthen their supervision and control over the management of the enterprise and create effective incentive constraints on the entrepreneur. This supervisory control by venture capital on the management of the company services can urge them to actively pursue technological innovation and increase the amount of capital invested in the firm for R&D, which ultimately help to improve the innovation performance of the company. Therefore, hypotheses are made:

*H2a*: The shareholding of venture capital institutions will have a catalytic effect on the innovation performance of enterprises.

#### 3.2.2. The impact of venture capital’s dispatching directors and occupying corporate board seats on the innovation performance of enterprises

Firstly, the board of directors is the most important decision-making and supervisory body in the corporate governance structure. Venture capitalists will often try to occupy seats on the board of directors of the company, hold decision-making power on important corporate matters, strengthen their status and role on the board of directors, improve their level of monitoring of the company, and improve the corporate governance structure. Venture capital give technical advice and guidance in the process of corporate innovation, etc. (Proksch et al., 2016), enhancing the positive effect of venture capital to the innovation efficiency of enterprises (Duan and Chen, 2020).

Secondly, by sending directors to the board of directors of enterprises, venture capital can help the board of directors better evaluate innovation-related policies by their high reputation, management experience, and network of relationships, and can also optimize R&D and innovation activities, guide enterprises hire more external independent directors with professional skills, enhance the professionalism and independence of the board of directors (Xiong and Gui, 2018), improve the decision-making level of important matters of the board of directors, and improve the strength of the board of directors to support enterprise innovation, help enhance the ability of enterprises in innovation and better realize the value-added of enterprise innovation.

Reasonable psychological expectations are key to the success of venture capital. Due to psychological bias, when investors make investment expectations based on inadequate and inaccurate information, they cannot effectively analyze the information, so their expectations about the future are difficult to completely conform to the actual situation, which directly leads to behavioral bias in venture capital. Faced with this problem, venture capital have tightened their grip on the dynamic economic information of start-up companies by sending directors and gaining seats on corporate boards to strengthen their oversight and control over corporate boards. Participation of venture capital in corporate governance can enhance their willingness to provide value-added services to companies, reduce the irrational part of investor expectations, and thus help companies make better entrepreneurial decisions, improve corporate governance, and enhance innovation efficiency. Therefore, hypotheses are made:

*H2b*: Venture capital institutions that sending directors and occupying seats on corporate boards will promote the innovation performance of enterprises.

### 3.3. Venture capital shareholding, dispatching directors, venture capital’s tolerance for innovation failure, innovation performance of enterprises

Failure tolerance refers to the ability of venture capital to tolerate corporate innovation failures and take risks. Equity financing will affect the technological innovation of enterprises because they can tolerate the failure risk caused by enterprise technological innovation. As a way of equity financing, venture capital by holding corporate shares and dispatching directors, not only invests in enterprises, and provides value-added services such as management supervision, but also effectively reduces the technical and management risks existing in the research and development process by bearing the possible failures of enterprises in the innovation process, and ultimately can bring positive impact on the innovation performance of enterprises.

(1) Due to the short establishment time of innovative and entrepreneurial enterprises and the relatively high degree of asset specialization, to form effective incentives and constraints for entrepreneurs, alleviate the risk of entrapping venture capital caused by entrepreneurs intervening in enterprises when they disagree with venture capital, venture capital avoid investment risks while reducing investment uncertainty by holding enterprise shares and sending directors to occupy board seats of enterprises.

By holding the equity of the enterprise and sending directors to occupy the seats on the board of directors of the enterprise, venture capital institutions determine to a certain extent their tolerance for technological innovation failures of the enterprise and the support for the company’s R&D investment. If the venture capital has a high shareholding ratio and sends directors to the board of directors of the enterprise, it can enhance its control over the enterprise, choose to actively participate in corporate governance, and will actively participate in the formulation of major business decisions, strategic guidelines and innovation decisions of the enterprise, realize the convergence of its interests with entrepreneurs, have longer-term support and tolerance for enterprises, have a stronger tolerance for the failure of enterprise R&D and innovation, and have a higher tolerance for the failure of enterprise technological innovation. It will choose to support enterprises to carry out technological innovation activities for a long time.

(2) On the one hand, firms are more likely to establish a culture of fault tolerance as an important part of the firm’s innovation culture if venture capitalists are tolerant of possible technological innovation failures of the firm. This will influence entrepreneurs’ attitudes towards innovation failures (Tian and Wang, 2014), raise the level of the risk appetite of entrepreneurs, make them willing to tolerate the high risks associated with technological innovation and invest more in R&D, support technical staff in their efforts to conduct technological trial and error (Zhang et al. 2021), which can reduce the cost of technological trial and error and the risks involved in the commercialization of innovative products, which will ultimately improve the innovation efficiency of enterprises. On the other hand, tolerance of innovation failure will strengthen venture capital’s belief in investment of in enterprises, and help them get more funds from the outside, reducing the possibility of innovation failure caused by financing constraints in the process of carrying out innovation activities.

On the one hand, venture capital send a positive signal to the External capital market by holding the equity of enterprises, the quality of the enterprise is certified, so that more external investors can be attracted to enter. This measure can reduce the possibility of technological innovation failure due to capital constraints, improve the entrepreneur’s appetite for risk, as well as the willingness to innovate, and realize the convergence of interests between it and venture capital, which can promote venture capital to support and tolerate enterprise technological innovation for a longer time, and the tolerance for enterprise innovation failure will be higher. It will strengthen supervision and control, and urge enterprises to accelerate innovation output and increase the value added of enterprise innovation. On the other hand, venture capital send directors to the board of directors of enterprises, implement supervision over the board of directors of enterprises, enhance the professionalism and independence of corporate boards, reduce the uncertainty they face when investing, improve their tolerance for technological innovation failures of enterprises, and improve their ability to bear various risks of enterprises. This can urge enterprises to carry out long-term and continuous technology research and development, improve their support for enterprise innovation, optimize the relevant processes in the project innovation process, and ultimately improve the innovation efficiency of enterprises.

The psychological capital of venture capital can increase their failure tolerance for the innovation activities of enterprises, which can then have a positive impact on the innovation performance of enterprises. The prospect, perception, concern and incentive degree of venture capital towards enterprises affect and influence entrepreneurs to varying degrees, transform their innovation awareness, and promote the consistency of enterprise values with venture capital’ risk preference and innovation willingness, thus increasing the value added of enterprises’ innovation. Luthans and Avey et al. (2009) found that it is necessary to improve the psychological capital of entrepreneurial institutional investors. The four dimensions of psychological capital, namely, confidence, hope, optimism, and perseverance, are all positive psychological states, which can improve the cognitive level of venture capital on the expectation of enterprise development prospects, enhance the tolerance of venture capital to the failure of innovation of enterprises, and enhance their willingness to provide value-added services for enterprises. This will not only help the company to build a fault-tolerant culture, but also help the company to obtain additional funding from outside sources and reduce innovation failures caused by financing constraints in innovation activities. Therefore, hypotheses are formulated.

*H3*: The tolerance of venture capital to innovation failure plays an mediating role between its shareholding ratio, dispatched directors and enterprise innovation performance.

### 3.4. The moderating effect of joint investment on the relationship between venture capital failure tolerance and the innovation performance of enterprises

Joint investment is often used as an investment strategy by venture capitalists because of the amount of capital required for corporate R&D and the high risk of innovation failure in the early stages of a business. Joint investment among venture capital institutions can provide enterprises with multiple financing channels, which can guarantee enterprises in the process of R&D and can also prevent the cash flow of the company from being affected so that the company has more capital This allows the company to invest in technological innovation. At the same time, the strategy of joint investment is conducive to diversifying the risks of the portfolio and balancing the risks between the various portfolios through phased investment; Venture capital institutions can also share resources and complementary professional skills among themselves by attracting other investment institutions to jointly invest, and share risks. Therefore, if they choose to make a joint investment, it will reduce the investment risks faced by venture capital, and venture capital can also obtain more assets with complementary nature (Wang and Zhou, 2017). Then, the tolerance of venture capital institutions for the failure of enterprises’ R&D activities will also be enhanced, and their tolerance for innovation risks will also increase, and they will provide long-term stable and sufficient funds for innovation investment, encourage enterprises to carry out riskier innovation projects, support enterprises to increase their R&D funds, and bring greater incentives for enterprises to innovate (Tian and Wang, 2014), which will ultimately help enterprises to be able to achieve more breakthroughs in science and technology innovation (Lu et al., 2017) and improve their innovation performance. Therefore, hypotheses are made.

*H4*: The joint investment strategy has a positive moderating effect on the relationship between the failure tolerance of venture capital institutions and the innovation performance of enterprises, and the more joint investment is chosen, the stronger the tolerance of venture capital to innovation failure will promote the innovation performance of enterprises.

### 3.5. The moderating effect of geographical proximity on the relationship between venture capital failure tolerance and the innovation performance of enterprises

Geographical proximity is important in venture capital investment decisions, with a preference for proximity to mitigate the uncertainty of the investment environment. If you choose local companies to invest nearby, more frequent face-to-face communication can take place between venture capital and companies, and both parties can be able to reduce transportation costs and time costs (Dong et al., 2019) and improve the efficiency, quality of mutual information exchange. At the same time, choose to invest closely, venture capital will face fierce competition, to obtain high-quality innovation projects, their attitude towards the innovation failure of enterprises will be more tolerant, tolerance for innovation failure will be higher, it will rely on its own professional investment experience, technology, network resources, for enterprises to carry out technological innovation to provide professional knowledge, strategic consulting, help enterprises formulate technology research and development, product development policies and strategies. In summary, with the enhancement of the ability of venture capital to take risks of enterprises, the tolerance of enterprises’ innovation failures has increased, in this case, frequent and positive contact and communication with entrepreneurs, improve the degree of the risk appetite of entrepreneurs, to promote its initiative in enterprise innovation, and ultimately improve the innovation performance of enterprises. Therefore, hypotheses are made:

*H5*: Geographical proximity has a positive moderating effect on the relationship between the failure tolerance of venture capital institutions and the innovation performance of enterprises, and if the close investment is chosen, the more tolerance of venture capital to innovation failure will promote the innovation performance of enterprises.

## 4. Study design

### 4.1. Data sources and sample selection

This paper selects all financing events and listed companies from the CVSource database from January 1, 2000, to December 31, 2019. Depending on the needs of the study, the sample was screened using the following criteria: (1) the CVSource database was used to find the financing data of listed companies from 2000 to 2019,define whether the company accepts the investment of venture capital according to the type of investment institutions; obtain data on VC shareholding ratio and joint investment scale; obtain data on enterprise characteristics; (2) Obtain data on various patent applications of the company from the State Intellectual Property Office and Baiteng.com through the manual collection; (3) Determine whether the board members are dispatched by venture capital and the investment institution to which they belong based on the personal characteristics and resumes of the company’s board members disclosed in the Guotai An database (CSMAR); (4) Find the deregistered company from the list of financing events in the CVSource database; on this basis, the data of VC failure tolerance is calculated; (5) Combine relevant data containing the same venture capital institution in the same investment round; (6) Delete samples with missing or missing data. According to the above standard processing, the final 5,119 company-year observations of VC investment were obtained.

### 4.2. Variable description and definition

#### 4.2.1. Explained variables

*Corporate innovation performance*. At this point, there is no unified standard for measuring the innovation performance of enterprises in academia, since the current level of information disclosure on innovation input by listed companies in China is not high, and, the number of patent applications selected can measure the effect of corporate innovation activities, which takes into account the corporate innovation input and reflects the efficiency (Chen et al., 2017). The granting of a company’s patent is, moreover, affected by the timing of granting as well as external uncertainties, among other factors. Therefore, drawing on studies by other scholars (Chemmanur et al., 2014), this paper uses the total number of patent applications (Patent) (including the number of inventions, utility model, and design patent applications) with a lag of 1 year to measure the performance of corporate innovation. Among the three types of patents mentioned above, invention patents contain more technological elements and are more innovative than the other two types of patents. Therefore, this paper also uses the number of invention patents filed with a one-year lag (Innovation) to measure the technological innovation performance of firms.

#### 4.2.2. Explanatory variables

(1) *VC Involvement*. To study the impact of whether venture capital enters the enterprise and its participation on the innovation performance of the enterprise, this paper sets a dummy variable for venture capital participation, which has a value of “1” if the enterprise receives funds from venture capital institutions in the year of the transaction, and “0” otherwise.

To study the mechanism of VC influencing the innovation performance of enterprises, this paper further uses the two variables of VC shareholding ratio and VC sending directors to the board of directors to measure the impact of VC on the innovation performance of enterprises by mastering control rights of the enterprise.

(2) *VC Share*: According to the data of financing events disclosed by CVSource database, measure the equity ratio of VC in financing events.

(3) *VC Accredited Directors*: After VC invests in the enterprise, before exiting the enterprise, if the venture capital sends directors to the board of directors of the enterprise in that year and has the decision-making power on important matters, the value of this variable is “1”; otherwise, the value of the variable is “0.”

#### 4.2.3. Mediation variables

*Tolerance*: Refers to the extent to which VCs tolerate the failure of technological innovation of a company. This paper uses the investment horizon of a VC between its initial investment and its decision to terminate its investment in an ultimately failed project to measure its failure tolerance (Tian and Wang, 2014). The duration of the VC in the firm that eventually fails is a weighted average, with the weight being the amount of their investment in that firm as a proportion of their total investment in that year.

#### 4.2.4. Moderating variables

(1) *Syndication*. This is measured by the number of VCs investing in a particular venture.

(2) *Distance*. The natural logarithm of the number of train miles between the VC and the enterprise is used to measure.

#### 4.2.5. Control variables

(1) *Stage*: set the dummy variable, that is, if the enterprise is in the early development stage (including the seed stage, and development stage) when receiving investment, the variable takes the value of “1,” otherwise, the variable takes the value of “0.”(2) *Industry*. According to the industry-level classification in the Qingke database, the industries to which enterprises belong are divided into five categories: broad IT, biotechnology/health, clean technology, services, and tradition. Since the broad IT industry has higher requirements for enterprise innovation than other industries, the technical content is higher, and the difficulty of enterprise innovation will be greater, to better study the impact of venture capital on enterprise innovation performance, this paper focuses on whether the broad IT industry will have an impact on enterprise innovation performance. Consequently, a dummy variable is set to “1” if the VC invests in a company in the broad IT industry, and “0” otherwise. (3) *GDP Growth Rate*. This paper also introduces the variable representing the year of investment into the regression to control for the fixed effects of the year of investment.

### 4.3. Model building

(1) To test hypotheses H1 and H2a-b, the total number of Patent applications (Patent) and the number of invention patent applications (Innovation) are taken as explained variables to study the influence of venture capital participation, shareholding ratio, dispatched directors on its innovation performance. The following model was set up for testing.


(1) $$\begin{array}{l} \mathrm{Patent}/{\mathrm{Innovation}}_{\mathrm{it}+1}=\alpha_{0}+\alpha_{1}{\mathrm{VCInvolvement}}_{\mathrm{it}}\\ \;+\alpha_{2}{\mathrm{Stage}}_{\mathrm{it}}+\alpha_{3}{\mathrm{GDPGrowthRate}}_{\mathrm{it}}\\ \;+\alpha_{4}{\mathrm{IT}}_{\mathrm{it}}++\gamma_{t}+\varepsilon_{\mathrm{it}} \end{array}$$

(2) $$\begin{array}{l} \mathrm{Patent}/{\mathrm{Innovation}}_{\mathrm{it}+1}=\beta_{0}+\beta_{1}\mathrm{VCShare}/{\mathrm{Accredited Directors}}_{\mathrm{it}}\\ \;+\beta_{2}{\mathrm{Stage}}_{\mathrm{it}}+\beta_{3}{\mathrm{GDPGrowthRate}}_{\mathrm{it}}\\ \;+\beta_{4}{\mathrm{IT}}_{\mathrm{it}}++\gamma_{t}+\varepsilon_{\mathrm{it}} \end{array}$$

(2) Firstly, to test H3, the Tolerance of venture capital to innovation failure is taken as the explained variable to study the impact of venture capital shareholding ratio and dispatched directors on the tolerance of venture capital failure, and the following model is set for testing.


(3) $$\begin{array}{l} \mathrm{VC}\;{\mathrm{Tolerance}}_{\mathrm{it}}=\gamma_{0}+\gamma_{1}\mathrm{VCShare}/{\mathrm{Accredited Directors}}_{\mathrm{it}}\\ \;+\gamma_{2}{\mathrm{Stage}}_{\mathrm{it}}+\gamma_{3}{\mathrm{GDPGrowthRate}}_{\mathrm{it}}\\ \;+\gamma_{4}{\mathrm{IT}}_{\mathrm{it}}++\gamma_{t}+\varepsilon_{\mathrm{it}} \end{array}$$

Secondly, the total number of Patent applications (Patent) and the number of invention patent applications (Innovation) are taken as explained variables to study the impact of venture capital shareholding ratio, dispatched directors, and failure tolerance of venture capital on enterprise innovation performance. The following model is set for the test.


(4) $$\begin{array}{l} \mathrm{Patent}/{\mathrm{Innovation}}_{\mathrm{it}+1}=\phi_{0}+\phi_{1}\mathrm{VC}\;{\mathrm{Tolerance}}_{\mathrm{it}}\\ \;+\;\phi_{2}{\mathrm{Stage}}_{\mathrm{it}}+\phi_{3}{\mathrm{GDPGrowthRate}}_{\mathrm{it}}\\ \;+\phi_{4}{\mathrm{IT}}_{\mathrm{it}}++\gamma_{t}+\varepsilon_{\mathrm{it}} \end{array}$$

(5) $$\begin{array}{l} \mathrm{Patent}/{\mathrm{Innovation}}_{\mathrm{it}+1}=\mu_{0}+\mu_{1}\mathrm{VCShare}/{\mathrm{Accredited Directors}}_{\mathrm{it}}\\ \;+\mu_{2}\mathrm{VC}\;{\mathrm{Tolerance}}_{\mathrm{it}}+\mu_{3}{\mathrm{Stage}}_{\mathrm{it}}\\ \;+\mu_{4}{\mathrm{GDPGrowthRate}}_{\mathrm{it}}+\mu_{5}{\mathrm{IT}}_{\mathrm{it}}++\gamma_{t}+\varepsilon_{\mathrm{it}} \end{array}$$

In the above regression model, The patent represents the total number of patent applications of the enterprise. Considering that the enterprise has not applied for patents in some years, the logarithm of the number of patent applications will be missing. In this paper, the total number of patent applications is added by 1, and then the logarithm is taken. Innovation represents the number of invention applications. In the model, add 1 to the number of invention patent applications to take the logarithm. Since the number of patent applications is a non-negative integer with a discrete distribution, this paper also uses the negative binomial regression model (NBR) for empirical test. $\gamma_{t}$ in Equations (1)–(5) represents the fixed effect existing in the investment year, while $\varepsilon_{it}$ represents the random disturbance term.

## 5. Empirical results and analysis

### 5.1. Descriptive statistics and correlation analysis

Table 1 lists the results of a simple descriptive statistical analysis of each of the main variables. The average number of patent and invention patent applications is 20.289 and 7.052 respectively, indicating that after receiving VC funds, enterprises actively carry out technological innovation activities; the average values of the proportion of equity held by VC and the proportion of directors sent to the board of directors are 3.87% and 0.169% respectively, indicating that the proportion of equity held by VC and the proportion of directors sent to the board of directors are relatively small. Different types of VC have great differences in terms of joint investment scale and geographical proximity. To avoid the influence brought by extreme values, this paper winsorizes all variables at 5% quantile before starting the empirical study, to avoid the problem of multicollinearity caused by the introduction of explanatory variables, mediating variables, and moderating variables.

**Table 1:** Descriptive statistics of main variables and Pearson correlation analysis of the main variables.

	Observations	Mean value	Standard Deviation	VC Share	Accredited Directors	Patent	Innovation	VC Tolerance	Stage	IT	GDP
VC Share	4,520	3.870	4.253	1	0.356^**^	0.469^**^	0.575^**^	0.268^**^	0.057^**^	0.039^*^	−0.028
Accredited Directors	5,119	0.169	0.375	0.356^**^	1	0.136^**^	0.180^**^	0.104^**^	0.015	0.006	−0.031
Patent	5,119	20.289	27.187	0.469^**^	0.136^**^	1	0.867^**^	0.217^**^	−0.061^**^	−0.076^**^	−0.033^*^
Innovation	5,119	7.052	10.061	0.575^**^	0.180^**^	0.867^**^	1	0.197^**^	−0.066^**^	0.017	−0.027
VC Tolerance	2,848	6.503	3.042	0.268^**^	0.104^**^	0.217^**^	0.197^**^	1	0.192^**^	0.044^*^	0.033
Stage	5,119	0.4500	0.5	0.057^**^	0.015	−0.061^**^	−0.066^**^	0.192^**^	1	0.063^**^	−0.002
IT	5,119	0.388	0.487	0.039^*^	0.006	−0.076^**^	0.017	0.044^*^	0.063^**^	1	−0.049^**^
GDP	5,119	1.663	2.096	−0.028	−0.031	−0.033^*^	−0.027	0.033	−0.002	−0.049^**^	1

**, * They are significant at the level of 0.01 and 0.05, respectively.

To compare whether enterprises with or without VC participation have significant differences in patent and other aspects, this paper adopts the mean value test (see Table 2 for the results). The results show that VC participation can not only provide enterprises with the funds needed for innovation, but also provide enterprises with professional knowledge and strategic consultation in technological innovation, and help enterprises improve their innovation ability and performance.

**Table 2:** Mean test.

Variable	VC involvement (*N* = 3,646)	NO VC involvement (*N* = 1,473)	T-test of sample difference
Patent	21.891	19.798	2.092^**^
Innovation	9.058	5.560	3.498^***^
Stage	0.529	0.427	0.102^***^
IT	0.392	0.377	0.015
GDP	1.668	1.652	0.015

***,**,*They are significant at the level of 0.01 and 0.05, respectively.

Table 1 shows the correlation coefficients between the VC shareholding ratio, dispatched directors, VC failure tolerance, and enterprise patents. The results show that the VC shareholding ratio and dispatched directors have a significant positive impact on the total number of patent applications and the number of invention patent applications of enterprises, that is, the higher the shareholding ratio, the dispatched directors to the board of directors of enterprises, VC will send a positive signal about the enterprise’s innovation ability to the external capital market, at this time, external investors will choose to invest more capital in the enterprise. It is helpful for enterprises to spend more funds on technology research and development, to improve their innovation performance. In addition, both VC shareholding ratio and dispatched directors have a significant positive impact on their tolerance for innovation failure, that is, the higher VC shareholding ratio and dispatched directors to the board of directors of enterprises can enhance their control over enterprises, and have a higher tolerance for technological innovation failure.

### 5.2. Impact of venture capital participation on enterprise innovation performance

In this part, multivariate regression and negative binomial regression models are used, respectively. After controlling variables such as enterprise development stage, IT industry, and GDP growth rate, as well as the fixed effect existing in the investment year, the influence of VC participation on the total amount of patent applications and invention patent applications of enterprises is studied, respectively. The results are shown in Table 3.

**Table 3:** The impact of venture capital involvement on enterprise innovation performance.

Variable	Patent		Innovation	
	OLS	NBR	OLS	NBR
	Model 1	Model 2	Model 3	Model 4
VC involvement	0.200^***^ (0.035)	0.126^***^ (0.041)	0.503^***^ (0.036)	0.511^***^ (0.040)
Stage	−0.131^***^ (0.031)	−0.078^**^ (0.036)	−0.226^***^ (0.033)	−0.177^**^ (0.035)
IT	−0.212^***^ (0.032)	−0.210^***^ (0.038)	−0.007 (0.034)	−0.009 (0.036)
GDP	−0.024^***^ (0.007)	−0.031^***^ (0.008)	−0.012 (0.008)	−0.012^***^ (0.008)
Constant	2.437^***^ (0.036)	3.132^***^ (0.042)	1.158^***^ (0.038)	1.807^***^ (0.042)
Year dummy	Control	Control	Control	Control
Observations	5,115	5,116	5,115	5,116
Adj *R*-squared	0.018		0.041	
*F*	25.007		55.934	
Log pseudolikelihood		−20,825.66		−15,967.834

The brackets are standard errors, and ***, ** and * are significant at the level of 0.01, 0.05 and 0.1, respectively.

Models 1–2 and 3–4, respectively, show the impact of VC participation on the total amount of patent applications and the number of invention patent applications. At the significance level of 1%, VC participation will have a positive impact on the total number of patent applications and invention patent applications of enterprises. The results show that, VC can solve the problems of insufficient R&D funds by providing the required funds to enterprises engaged in R&D activities; VC provides professional knowledge and technical guidance for enterprises to carry out technological innovation, helps enterprises to innovate by obtaining valuable technologies and resources from enterprises in the same industry. Finally, improve the performance of enterprise innovation. This also proves Hypothesis H1.

Among the control variables, enterprise development stage, IT industry, and GDP growth rate have a significant negative impact on enterprise innovation performance.

### 5.3. Impact of venture capital shareholding ratio and directors dispatched on enterprise innovation performance

This part uses multivariate regression and negative binomial regression model respectively, to study the impact of the proportion of equity held by VC and the dispatch of directors on the total number of patent applications and the number of invention patent applications of enterprises. The results are presented in Table 4.

**Table 4:** Impact of VC shareholding and dispatched directors on enterprise innovation performance.

Variable	Patent				Innovation			
	OLS	NBR	OLS	NBR	OLS	NBR	OLS	NBR
	Model 5	Model 6	Model 7	Model 8	Model 9	Model 10	Model 11	Model 12
VC share	0.028^***^ (0.004)	0.017^***^ (0.028)			0.121^***^ (0.003)	0.104^***^ (0.002)		
Accredited Directors			0.385^***^ (0.046)	0.319^***^ (0.048)			0.623^***^ (0.056)	0.500^***^ (0.054)
Stage	−0.128^***^ (0.039)	−0.189^**^ (0.087)	−0.131^***^ (0.037)	−0.049 (0.041)	−0.196^***^ (0.040)	−0.048 (0.049)	−0.192^***^ (0.044)	−0.049 (0.050)
IT	−0.199^***^ (0.040)	−0.323^***^ (0.080)	−0.171^***^ (0.037)	−0.153^***^ (0.043)	0.013 (0.041)	0.064 (0.051)	0.055 (0.045)	0.090^*^ (0.052)
GDP	−0.025^***^ (0.009)	−0.030^***^ (0.011)	−0.017^**^ (0.009)	−0.022^**^ (0.010)	−0.009 (0.009)	−0.004 (0.012)	−0.013 (0.011)	−0.018 (0.012)
Constant	2.529^***^ (0.039)	3.524^***^ (0.100)	2.536^***^ (0.035)	2.536^***^ (0.035)	1.002^***^ (0.040)	1.652^***^ (0.047)	1.527^***^ (0.042)	2.376^***^ (0.047)
Year dummy	Control	Control	Control	Control	Control	Control	Control	Control
Observations	3,201	3,202	3,643	3,644	3,157	3,158	3,643	3,644
Adj *R*-squared	0.023		0.028		0.336		0.037	
*F*	19.719		27.089		399.764		36.073	
Log pseudolikelihood		−13,791.45		−14,926.055		−10,414.851		−12,583.93

The brackets are standard errors, and ***, **, and * are significant at the level of 0.01, 0.05, and 0.1, respectively.

Models 5–6 and 9–10, respectively, show the influence of the VC shareholding ratio on the total amount of patent applications and invention patent applications. At the significance level of 1%, the proportion of equity held by VC has a positive impact on the total number of patent applications and the number of invention patent applications. The results show that the higher the shareholding ratio, the more capital, and value-added services it can provide to enterprises. It can provide enterprises with more technical support and guidance for technology research and development and new product development, and the entrepreneurs will also be more willing to innovate, and finally promote the improvement of enterprise innovation performance. This also proves that hypothesis H2a.

Models 7–8 and 11–12 show the impact of VC sending directors to the board of directors on the total number of patent applications and invention patent applications, respectively. At the significance level of 1%, VC sending directors to the board of directors has a positive impact on the total number of patent applications and invention patent applications. The results show that, after dispatching directors to enterprises, VC will strengthen its position and role in the board of directors through hold seats on the board of directors, and give technical advice and guidance in the process of enterprise innovation, rationally allocating various resources used in the process of enterprise innovation, and finally improving the performance of enterprise innovation. This also proves Hypothesis H2b.The influence of each control variable on enterprise innovation performance is the same as that of the full sample.

### 5.4. Mediating effect of venture capital’s tolerance for innovation failure on the relationship between its shareholding, directors dispatch and enterprise innovation performance

To test the mediating effect of the tolerance of venture capital on the relationship among their shareholding ratio, dispatched directors, and enterprise innovation performance, this part test whether the coefficient of variables in the regression model is significant, that is, if *β*_1_ in Equation 2 is significant, *γ*_1_ in Eqaution 3 is significant, and μ_2_,μ_1_in Equation 5 is significant; also *μ*_1_ less than *β*_1_, if the coefficient is reduced, VC’s tolerance for innovation failure will play a part of mediation role; If *μ*_1_ not significant, *μ*_2_ significant, VC plays a complete mediation role in the tolerance of innovation failure.

Firstly, Models 13 and 16 in Table 5 and Models 23 and 26 in Table 6 verify that VC’s shareholding ratio and sending directors to the board of directors have an impact on their tolerance for innovation failure. At the significance level of 1%, the proportion of equity held by the VC and the dispatched directors, respectively, have a significantly positive impact on its failure tolerance. The results show that: If the shareholding ratio is high and the directors are sent to the board of directors of enterprises, VC will realize the convergence of its interests with entrepreneurs. They have a higher tolerance for technological innovation failure.

**Table 5:** Impact of venture capital shareholding and failure tolerance on enterprise innovation performance.

Variable	VC Tolerance	Patent		VC Tolerance	Innovation		Patent		Innovation	
	OLS	OLS	NBR	OLS	OLS	NBR	OLS	NBR	OLS	NBR
	Model 13	Model 14	Model 15	Model 16	Model 17	Model 18	Model 19	Model 20	Model 21	Model 22
VC share	0.105^***^ (0.014)			0.114^***^ (0.010)			0.011^***^ (0.006)	0.016^***^ (0.004)	0.114^***^ (0.004)	0.093^***^ (0.003)
VC Tolerance		0.054^***^ (0.009)	0.092^***^ (0.011)		0.094^***^ (0.009)	0.112^***^ (0.009)	0.069^***^ (0.009)	0.106^***^ (0.012)	0.044^***^ (0.009)	0.082^***^ (0.009)
Stage	1.264^***^ (0.126)	−0.068 (0.049)	−0.225^**^ (0.098)	0.956^***^ (0.132)	−0.227^***^ (0.058)	−0.181^***^ (0.060)	−0.092^*^ (0.052)	−0.258^**^ (0.108)	−0.193^***^ (0.052)	−0.109^**^ (0.055)
IT	0.057 (0.129)	−0.117^**^ (0.049)	−0.232^***^ (0.090)	0.060 (0.136)	0.139^**^ (0.058)	0.178^***^ (0.058)	−0.141^***^ (0.052)	−0.225^**^ (0.099)	0.099^*^ (0.053)	0.170^***^ (0.056)
GDP	0.033 (0.029)	−0.007 (0.011)	−0.019 (0.015)	0.029 (0.031)	0.002 (0.013)	−0.013 (0.013)	−0.010 (0.012)	−0.031^**^ (0.014)	0.006 (0.012)	−0.001 (0.012)
Constant	5.807^***^ (0.119)	2.364^***^ (0.065)	3.071^***^ (0.107)	5.506^***^ (0.128)	1.187^***^ (0.072)	1.887^***^ (0.073)	2.237^***^ (0.072)	2.924^***^ (0.118)	0.845^***^ (0.069)	1.251^***^ (0.067)
Year dummy	Control	Control	Control	Control	Control	Control	Control	Control	Control	Control
Observations	1,883	2,179	2,180	1,937	2,297	2,298	1,883	1,884	1,937	1,938
Adj *R*-squared	0.089	0.018		0.095	0.046		0.035		0.348	
*F*	46.929	11.255		52.069	28.403		14.531		207.781	
Log pseudolikelihood			−9,735.939			−8,251.332		−8,428.775		−6,682.154

The brackets are standard errors, and ***, **, and * are significant at the level of 0.01, 0.05, and 0.1, respectively.

**Table 6:** Impact of dispatched directors and failure tolerance of venture capital on enterprise innovation performance.

Variable	VC Tolerance	Patent		VC Tolerance	Innovation		Patent		Innovation	
	OLS	OLS	NBR	OLS	OLS	NBR	OLS	NBR	OLS	NBR
	Model 23	Model 24	Model 25	Model 26	Model 27	Model 28	Model 29	Model 30	Model 31	Model 32
Accredited Directors	0.432^***^ (0.149)			0.826^***^ (0.163)			0.309^***^ (0.059)	0.266^***^ (0.058)	0.582^***^ (0.072)	0.451^***^ (0.065)
VC Tolerance		0.054^***^ (0.009)	0.092^***^ (0.011)		0.094^***^ (0.009)	0.112^***^ (0.009)	0.051^***^ (0.008)	0.073^***^ (0.007)	0.086^***^ (0.009)	0.108^***^ (0.009)
Stage	1.526^***^ (0.119)	−0.068 (0.049)	−0.225^**^ (0.098)	1.182^***^ (0.128)	−0.227^***^ (0.058)	−0.181^***^ (0.060)	−0.068 (0.049)	−0.025 (0.050)	−0.220^***^ (0.057)	−0.164^***^ (0.061)
IT	0.113 (0.122)	−0.117^**^ (0.049)	−0.232^***^ (0.090)	0.206 (0.131)	0.139^**^ (0.058)	0.178^***^ (0.058)	−0.114^**^ (0.048)	−0.076 (0.050)	0.144^**^ (0.057)	0.193^***^ (0.058)
GDP	0.050^*^ (0.028)	−0.007 (0.011)	−0.019 (0.015)	0.050^*^ (0.030)	0.002 (0.013)	−0.013 (0.013)	−0.007 (0.011)	−0.017 (0.011)	0.003 (0.013)	−0.010 (0.013)
Constant	5.866^***^ (0.107)	2.364^***^ (0.065)	3.071^***^ (0.107)	5.586^***^ (0.115)	1.187^***^ (0.072)	1.887^***^ (0.073)	2.320^***^ (0.066)	2.266^***^ (0.058)	1.121^***^ (0.072)	1.794^***^ (0.072)
Year dummy	Control	Control	Control	Control	Control	Control	Control	Control	Control	Control
Observations	2,179	2,179	2,180	2,297	2,297	2,298	2,179	2,180	2,297	2,298
Adj *R*-squared	0.075	0.018		0.048	0.046		0.030		0.072	
*F*	44.935	11.255		29.891	28.403		14.545		36.504	
Log pseudolikelihood			−9,735.939			−8,251.332		−9,232.265		−8,223.702

The brackets are standard errors, and ***, **, and * are significant at the level of 0.01, 0.05, and 0.1, respectively.

Secondly, through models 14–15 and 17–18 in Table 5 and 24–25 and 27–28 in Table 6, the influence of VC’s tolerance for innovation failure on enterprise innovation performance is studied. At the significance level of 1%, VC’s tolerance for innovation failure has a significant positive impact on the total number of patent applications and the number of invention patent applications. The results show that: Enterprises supported by VC who can tolerate technological innovation failure are more likely to establish a culture of tolerance for failure, which will affect entrepreneurs’ attitude towards innovation failure, improve their risk preference, make them willing to bear higher technological innovation risks, increase R&D investment, achieve the purpose of improving enterprise innovation efficiency.

Finally, Model 19–22 in Table 5 and Model 29–32 in Table 6 are used to study the mediating effect of VC’s tolerance for innovation failure on its shareholding ratio, dispatched directors, and enterprise innovation performance. The research results show that after the variable of VC failure tolerance is added when the total number of patent applications is taken as the explained variable, the coefficient of VC’s shareholding ratio decreases from 0.028 to 0.011, and the coefficient of VC’s dispatched directors decreases from 0.385 to 0.309. When the number of invention patent applications was taken as the explained variable, the coefficient of VC’s shareholding ratio and the coefficient of dispatched directors also decreased. The coefficient of VC’s shareholding ratio decreased from 0.121 to 0.114, and the coefficient of VC’s dispatched directors decreased from 0.623 to 0.582, and the regression coefficient was still significant. In the negative binomial regression model, VC’s shareholding ratio and the coefficient of sending directors also decreased. The results show that VC’s tolerance for innovation failure plays a partial mediating effect between its shareholding ratio, dispatched directors, and enterprise innovation performance and hypothesis H3 is verified.

To test the value of the mediating effect produced by VC’s innovation failure tolerance, this paper adopts the Process program. The direct effect, indirect effect, and total effect values between VC’s shareholding ratio dispatched directors (explanatory variable), VC’s tolerance for innovation failure Mediation variables, total patent applications and invention patent applications (explained variable) are estimated, and the results are shown in Table 7. The results show that when the total number of patent applications (number of invention patent applications) is taken as the explained variable, in the first stage, the influence coefficients of VC shareholding ratio and dispatched directors on VC failure tolerance are 0.105 and 0.432 (0.114 and 0.826), respectively, which have a significant impact on the results. In the second stage, in the case of VC shareholding and dispatching directors. The coefficients of the effects of VC failure tolerance on the total number of corporate patent applications (number of invention patent applications) were 0.069 and 0.051 (0.044 and 0.086), respectively, with significant results. That is, the direct effect coefficients of the VC shareholding ratio and dispatched directors on the total amount of patent applications (invention patent applications) are 0.012 and 0.309 (0.114 and 0.582) respectively. The indirect effects of the VC shareholding ratio and dispatched directors on the total amount of enterprise patent applications (invention patent applications) are 0.007 and 0.002 (0.005 and 0.071), respectively, and the results are significant. Therefore, the total effect coefficients of VC’s shareholding ratio and dispatched directors on the total amount of patent applications (invention patent applications) are 0.019 and 0.331 (0.119 and 0.653). The partial mediating effect of VC failure tolerance was verified, assuming that H3 passed the test.

**Table 7:** Direct, indirect, and total effects of VC shareholding and dispatched directors on enterprise innovation performance through their failure tolerance.

Variable	Stage		Effect		
	Phase I	Phase II	Direct effect	Indirect effect	Total effect
VC Share	0.105^***^ (0.114^***^)	0.069^***^ (0.044^***^)	0.012^**^ (0.114^***^)	0.007^***^ (0.005^***^)	0.019^***^ (0.119^***^)
VC Accredited Directors	0.432^**^ (0.826^***^)	0.051^***^ (0.086^***^)	0.309^***^ (0.582^***^)	0.002*** (0.071^***^)	0.331^***^ (0.653^***^)

In parentheses are the effect values corresponding to the invention patent as the explained variable，***, **, and *are significant at the level of 0.01, 0.05, and 0.1, respectively.

### 5.5. Moderating effect of characteristics of heterogeneous venture capital institutions

There is heterogeneity among different types of venture capital institutions, which are significantly different in joint investment strategy and geographical proximity, and these differences will affect the relationship between venture capital institutions’ tolerance of early innovation failure and enterprise innovation performance.

#### 5.5.1. Moderating effect of joint investment on the relationship between VC failure tolerance and enterprise innovation performance

To study the moderating effect of joint investment strategy on the relationship between VC’s failure tolerance and enterprise innovation performance, this paper divides the samples into groups and divides the samples into individual investment and joint investment samples according to the number of joint investment institutions. On this basis, regression analysis is carried out for VC in different samples. The relationship between innovation failure tolerance and innovation performance in the early stage is studied. The results are shown in Table 8.

**Table 8:** Regression results of the moderating effect of joint investment on the relationship between VC failure tolerance and enterprise innovation performance.

Variable	Patent				Innovation			
	Joint investment		Independent investment		Joint investment		Independent investment	
	OLS	NBR	OLS	NBR	OLS	NBR	OLS	NBR
	Model 33	Model 34	Model 35	Model 36	Model 37	Model 38	Model 39	Model 40
VC Tolerance	0.063^***^ (0.009)	0.085^***^ (0.008)	0.018 (0.020)	0.039^**^ (0.017)	0.100^***^ (0.010)	0.126^***^ (0.010)	0.074^***^ (0.023)	0.073^***^ (0.019)
Stage	−0.101^*^ (0.053)	−0.061 (0.055)	0.148 (0.131)	0.116 (0.125)	−0.230^***^ (0.062)	−0.192^***^ (0.067)	−0.152 (0.154)	−0.100 (0.147)
IT	−0.191^***^ (0.053)	−0.132^**^ (0.055)	0.314^**^ (0.130)	0.196 (0.120)	0.057 (0.063)	0.118^*^ (0.064)	0.674^***^ (0.161)	0.466^***^ (0.141)
GDP	−0.001 (0.012)	−0.017 (0.013)	−0.061^**^ (0.026)	−0.049^**^ (0.024)	0.013 (0.015)	−0.008 (0.016)	−0.072^**^ (0.033)	−0.056^**^ (0.025)
Constant	2.338^***^ (0.071)	2.756^***^ (0.069)	2.451^***^ (0.162)	2.902^***^ (0.141)	1.143^***^ (0.079)	1.800^***^ (0.082)	1.320^***^ (0.188)	2.218^***^ (0.169)
Year dummy	Control	Control	Control	Control	Control	Control	Control	Control
Observations	1,833	1,834	345	346	1,934	1,935	362	363
Adj *R*-squared	0.028		0.024		0.050		0.064	
*F*	14.180		3.097		26.314		7.184	
Log pseudolikelihood		−7,778.644		−1,460.631		−6,905.569		−1,354.134

The brackets are standard errors, and ***, **, and * are significant at the level of 0.01, 0.05 and 0.1, respectively.

Table 8 shows the moderating effect of joint investment strategy on the relationship between VC failure tolerance, total patent applications, and the number of invention patent applications. Models 33, 35 and 37, 39 show the regression coefficient between the tolerance of VC who choose joint investment and independent investment for innovation failure and the total number of enterprise patent applications (number of invention patent applications) are 0.063 and 0.018 (0.1 and 0.074), respectively, except that when the total amount of patent applications is taken as the explained variable, the coefficient of investment alone is not significant. In other cases, the regression coefficients were significant at the 1% level. The research results show that joint investment will reduce the investment risks faced by VC, VC’s tolerance of innovation failure will also increase, it will support enterprises to carry out high-risk R&D innovation, and bring more incentive effect to enterprise innovation. The above results show that the joint investment strategy has a positive moderating effect on the relationship between the VC’s tolerance of innovation failure in the early stage and the firm’s innovation performance This result confirms hypothesis H4.

#### 5.5.2. The moderating effect of geographical proximity on the relationship between VC failure tolerance and enterprise innovation performance

To study the moderating effect of geographical proximity on the relationship between VC’s failure tolerance and an enterprise’s innovation performance, this paper grouped the samples and divided them into remote and near-distance investment samples according to the mean value of geographical distance. On this basis, regression analysis was conducted on VC in different samples. The relationship between innovation failure tolerance and innovation performance is studied. The results are shown in Table 9.

**Table 9:** Regression results of the moderating effect of geographical proximity on the relationship between VC failure tolerance and enterprise innovation performance.

Variable	Patent				Innovation			
	Proximity investment		Long distance investment		Proximity investment		Long distance investment	
	OLS	NBR	OLS	NBR	OLS	NBR	OLS	NBR
	Model 41	Model 42	Model 43	Model 44	Model 45	Model 46	Model 47	Model 48
VC Tolerance	0.062^***^ (0.015)	0.055^***^ (0.012)	0.046^***^ (0.011)	0.038^***^ (0.009)	0.120^***^ (0.016)	0.082^***^ (0.012)	0.072^***^ (0.012)	0.051^***^ (0.008)
Stage	−0.121 (0.087)	−0.074 (0.058)	−0.023 (0.064)	−0.015 (0.040)	−0.203^*^ (0.104)	−0.086 (0.061)	−0.090 (0.069)	−0.035 (0.040)
IT	−0.139 (0.089)	−0.099 (0.060)	−0.098 (0.062)	−0.067^*^ (0.039)	0.141 (0.107)	0.060 (0.064)	0.006 (0.068)	−0.005 (0.040)
GDP	−0.002 (0.021)	0.002 (0.018)	−0.001 (0.014)	−0.002 (0.009)	−0.001 (0.025)	0.003 (0.021)	0.014 (0.015)	0.007 (0.009)
Constant	2.413^***^ (0.119)	2.967^***^ (0.096)	2.363^***^ (0.083)	3.034^***^ (0.060)	1.046^***^ (0.135)	2.127^***^ (0.101)	1.916^***^ (0.089)	2.797^***^ (0.062)
Year dummy	Control	Control	Control	Control	Control	Control	Control	Control
Observations	631	632	1,393	1,394	672	673	1,414	1,415
Adj *R*-squared	0.026		0.011		0.074		0.024	
*F*	5.141		4.980		14.369		9.682	
Log pseudolikelihood		−2,710.857		−5,953.438		−2,453.025		−5,850.723

The brackets are standard errors, and ***, **, and * are significant at the level of 0.01, 0.05 and 0.1, respectively.

Table 9 shows the moderating effect of geographical proximity on the relationship between VC failure tolerance, the total amount of patent applications, and the number of invention patent applications. Models 41, 43, 45 and 47 show that the regression coefficients of VC’s tolerance of innovation failure for short distance investment and long distance investment to the total number of enterprise patent applications (number of invention applications) are 0.062 and 0.046 (0.12 and 0.072), respectively, which are significant at the 1% level. The research results show that, in the case of close-range investment, VC will face fierce competition. To obtain high-quality innovation projects, VC will be more tolerant of possible technological innovation risks. VC will provide help for enterprise technology research and development and value creation. Finally, it helps enterprises achieve the purpose of improving innovation performance.

The above results show that geographical proximity has a positive moderating effect between VC’s tolerance for innovation failure of an enterprise and its innovation performance. This result confirms hypothesis H5.

## 6. Robustness analysis

### 6.1. Propensity score matching analysis

The above research shows that enterprises with venture capital participation, higher shareholding ratio, and sending directors to the board of directors have higher innovation performance. Considering that such results may be caused by strong innovation ability and high innovation performance of enterprises, The above influence of venture capital participation, shareholding and dispatched directors on enterprise innovation performance may come from the “selection effect” in advance, rather than the value-added effect brought by holding enterprise equity and sending directors afterwards. In order to better identify the “selection effect” and “value-added effect” of VC, the propensity score matching method (PSM) is used for analysis, and the nearest neighbor matching method is combined with the self-sampling method.

First of all, this paper studies the effects of VC’s shareholding ratio and the dispatch of directors on their tolerance for innovation failure and corporate innovation performance. The enterprises with higher than average VC holding ratio and sending directors were taken as the treatment group, and the enterprises with lower than average VC holding ratio and not sending directors were taken as the control group, and the control variables were taken as matching variables for propensity score matching analysis. The average processing effect results of VC’s shareholding ratio, dispatched directors on failure tolerance and enterprise innovation performance before and after matching are shown in Table 10. After matching, at the significant level of 1%, the total number of patent applications and invention patent applications of enterprises are different from 0.After matching, the mean value of total patent applications (number of invention patent applications) of the group with higher shareholding ratio and dispatched directors were 2.478 and 2.726 (2.574 and 2.042), respectively, and the average ATT treatment effect was 0.179 and 0.385 (1.479 and 0.601), respectively. In addition, both before and after matching, VC’s tolerance for innovation failure is different from 0. After matching, VC’s mean tolerance for innovation failure is 7.464 and 7.149, respectively, and ATT’s average treatment effect is 0.758 and 0.472, respectively. Compared with other VCS, those who hold a higher proportion of corporate equity and send directors to occupy the seats of the board of directors will have a higher tolerance for innovation failure, and the innovation performance of the enterprises they invest in will also be higher.

**Table 10:** Average treatment effect (ATT) of VC shareholding ratio, dispatched directors’ tolerance to failure, and enterprise innovation performance.

Variable	Sample	Average treatment effect of VC shareholding ratio on its failure tolerance and enterprise innovation performance					Average treatment effect of VC dispatched directors on their failure tolerance and enterprise innovation performance				
		Processing group	Control group	ATT	Standard error	*T* value	Processing group	Control group	ATT	Standard error	*T* value
Innovation	Before matching	2.574	1.128	1.447	0.062	23.32^***^	2.042	1.410	0.632	0.072	8.79^***^
	After matching	2.574	1.095	1.479	0.067	21.92^***^	2.042	1.442	0.601	0.075	8.03^***^
Patent	Before matching	2.478	2.399	0.078	0.053	1.48	2.726	2.325	0.400	0.058	6.89^***^
	After matching	2.478	2.297	0.179	0.058	3.08^***^	2.726	2.340	0.385	0.060	6.46^***^
VC Tolerance	Before matching	7.464	6.275	1.189	0.178	6.69^***^	7.149	6.695	0.454	0.202	2.24^**^
	After matching	7.464	6.706	0.758	0.198	3.82^***^	7.149	6.677	0.472	0.218	2.16^**^

Secondly, this paper uses PSM to match each VC that holds corporate equity and dispatches directors with a VC that is similar in other aspects but does not hold corporate equity and dispatches directors. On this basis, regression analysis is carried out. First, the enterprise development stage and industry attributes, as well as the year variables as matching variables; Secondly, the Logit model is used to estimate the probability of VC holding corporate equity and dispatching directors. Thirdly, for each VC holding corporate equity and dispatching directors, a VC with similar propensity score, without holding corporate equity and dispatching directors is matched; Finally, regression analysis was conducted again on the obtained paired samples, and the results were shown in Tables 11–13.

**Table 11:** Impact of VC shareholding and dispatched directors on enterprise innovation performance.

Variable	Patent				Innovation			
	OLS	NBR	OLS	NBR	OLS	NBR	OLS	NBR
	Model 49	Model 50	Model 51	Model 52	Model 53	Model 54	Model 55	Model 56
VC Share	0.030^***^ (0.004)	0.020^***^ (0.003)			0.121^***^ (0.003)	0.086^***^ (0.002)		
Accredited Directors			0.480^***^ (0.058)	0.292^***^ (0.036)			0.620^***^ (0.074)	0.323^***^ (0.040)
Stage	−0.117^***^ (0.042)	−0.064^**^ (0.026)	−0.222^***^ (0.059)	−0.128^***^ (0.037)	−0.202^***^ (0.043)	−0.145^***^ (0.028)	−0.277^***^ (0.075)	−0.130^***^ (0.041)
IT	−0.219^***^ (0.043)	−0.128^***^ (0.026)	−0.056 (0.060)	−0.037 (0.037)	0.010 (0.044)	−0.002 (0.029)	0.111 (0.076)	0.060 (0.042)
GDP	−0.026^**^ (0.010)	−0.016^**^ (0.007)	−0.022 (0.013)	−0.012 (0.009)	−0.004 (0.011)	−0.007 (0.007)	−0.022 (0.017)	−0.010 (0.010)
Constant	2.517^***^ (0.043)	3.120^***^ (0.034)	2.451^***^ (0.061)	3.081^***^ (0.046)	0.986^***^ (0.043)	1.891^***^ (0.037)	1.568^***^ (0.078)	2.541^***^ (0.052)
Year dummy	Control	Control	Control	Control	Control	Control	Control	Control
Observations	2,772	2,773	1,407	1,408	2,799	2,800	1,407	1,408
Adj *R*-squared	0.027		0.054		0.342		0.055	
*F*	20.040		21.095		364.224		21.466	
Log pseudolikelihood		−11,398.963		−5,828.795		−9,218.066		−5,107.579

The brackets are standard errors, and ***, **, and * are significant at the level of 0.01, 0.05, and 0.1, respectively.

**Table 12:** Impact of venture capital shareholding and failure tolerance on enterprise innovation performance.

Variable	VC Tolerance	Patent		VC Tolerance	Innovation		Patent		Innovation	
	OLS	OLS	NBR	OLS	OLS	NBR	OLS	NBR	OLS	NBR
	Model 57	Model 58	Model 59	Model 60	Model 61	Model 62	Model 63	Model 64	Model 65	Model 66
VC Share	0.108^***^ (0.015)			0.109^***^ (0.010)			0.016^***^ (0.006)	0.007^*^ (0.004)	0.116^***^ (0.004)	0.081^***^ (0.003)
VC Tolerance		0.072^***^ (0.010)	0.061^***^ (0.008)		0.111^***^ (0.011)	0.078^***^ (0.008)	0.067^***^ (0.010)	0.057^***^ (0.008)	0.044^***^ (0.009)	0.032^***^ (0.007)
Stage	1.288^***^ (0.138)	−0.068 (0.056)	−0.039 (0.037)	0.843^***^ (0.142)	−0.181^***^ (0.067)	−0.074^*^ (0.038)	−0.069 (0.056)	−0.037 (0.036)	−0.172^***^ (0.055)	−0.111^**^ (0.036)
IT	0.023 (0.142)	−0.133^**^ (0.056)	−0.097^***^ (0.037)	0.074 (0.148)	0.164^**^ (0.069)	0.080^**^ (0.040)	−0.140^**^ (0.056)	−0.099^***^ (0.037)	0.097^*^ (0.057)	0.019 (0.039)
GDP	0.041 (0.034)	−0.010 (0.013)	−0.007 (0.010)	0.043 (0.035)	0.011 (0.016)	0.010 (0.010)	−0.010 (0.013)	−0.007 (0.010)	0.005 (0.013)	−0.007 (0.010)
Constant	5.816^***^ (0.136)	2.253^***^ (0.078)	2.902^***^ (0.059)	5.558^***^ (0.139)	1.051^***^ (0.090)	2.157^***^ (0.067)	2.220^***^ (0.079)	2.895^***^ (0.058)	0.800^***^ (0.075)	1.824^***^ (0.055)
Year dummy	Control	Control	Control	Control	Control	Control	Control	Control	Control	Control
Observations	1,610	1,611	1,612	1,710	1,706	1,711	1,610	1,611	1,710	1,711
Adj *R*-squared	0.084	0.033		0.083	0.059		0.037		0.362	
*F*	37.735	14.841		39.530	27.676		13.405		194.865	
Log pseudolikelihood			−6,887.462			−6,290.641		−6,882.090		−5,939.336

The brackets are standard errors, and ***, **, and * are significant at the level of 0.01, 0.05, and 0.1, respectively.

**Table 13:** Impact of dispatched directors and failure tolerance of venture capital on enterprise innovation performance.

Variable	VC Tolerance	Patent		VC Tolerance	Innovation		Patent		Innovation	
	OLS	OLS	NBR	OLS	OLS	NBR	OLS	NBR	OLS	NBR
	Model 67	Model 68	Model 69	Model 70	Model 71	Model 72	Model 73	Model 74	Model 75	Model 76
Accredited Directors	0.618^***^ (0.198)			1.536^***^ (0.211)			0.318^***^ (0.076)	0.205^***^ (0.048)	0.541^***^ (0.094)	0.282^***^ (0.052)
VC Tolerance		0.033^**^ (0.013)	0.029^***^ (0.010)		0.107^***^ (0.014)	0.065^***^ (0.009)	0.027^**^ (0.013)	0.028^***^ (0.010)	0.088^***^ (0.014)	0.056^***^ (0.009)
Stage	1.969^***^ (0.200)	−0.071 (0.081)	−0.052 (0.052)	1.362^***^ (0.212)	−0.336^***^ (0.095)	−0.143^***^ (0.052)	−0.078 (0.080)	−0.060 (0.053)	−0.317^***^ (0.094)	−0.132^**^ (0.052)
IT	0.027 (0.205)	0.005 (0.079)	0.004 (0.050)	0.242 (0.218)	0.224^**^ (0.096)	0.118^**^ (0.054)	0.018 (0.078)	0.009 (0.050)	0.237^**^ (0.094)	0.124^**^ (0.053)
GDP	0.106^**^ (0.044)	−0.026 (0.017)	−0.018 (0.011)	0.099^**^ (0.047)	−0.030 (0.021)	−0.017 (0.012)	−0.028 (0.017)	−0.019 (0.011)	−0.032 (0.020)	−0.019 (0.012)
Constant	5.409^***^ (0.197)	2.588^***^ (0.100)	3.272^***^ (0.051)	4.698^***^ (0.208)	1.352^***^ (0.111)	2.432^***^ (0.073)	2.470^***^ (0.103)	3.072^***^ (0.077)	1.211^***^ (0.112)	2.344^***^ (0.075)
Year dummy	Control	Control	Control	Control	Control	Control	Control	Control	Control	Control
Observations	855	855	856	930	930	931	855	856	930	931
Adj *R*-squared	0.115	0.005		0.095	0.066		0.024		0.097	
*F*	28.860	2.023		25.395	17.337		5.160		21.017	
Log pseudolikelihood			−3,708.425			−3,515.168		−3,701.349		−3,503.909

The brackets are standard errors, and ***, **, and * are significant at the level of 0.01, 0.05, and 0.1, respectively.

(1) Models 49–50 and 53–54 in Table 11 and 51–52 and 55–56 in Table 11 show that, at the significance level of 1%, the proportion of equity held by VC and the number of directors dispatched by VC have a positive impact on the total number of patent applications and the number of invention patent applications of enterprises. The higher the proportion of VC shares, the more directors are dispatched to the board of directors of enterprises, ultimately help enterprises to achieve the purpose of improving innovation performance.

(2) Models 57 and 60 in Tables 12 and 67 and 70 in Table 13 show that, at the significance level of 1%, the proportion of equity held by VC and the dispatch of directors have a positive impact on its tolerance of innovation failure. If VC has a high proportion of shareholding and sends directors to the board of directors of enterprises, there will also be a higher tolerance for failure in technological innovation.

(3) Models 58–59 and 61–62 in Table 12 and 68–69 and 71–72 in Table 13 show that VC’s tolerance for innovation failure has a positive impact on the total number of patent applications and the number of invention patent applications under the significance level of 1%. If VC has a high tolerance for innovation failure, entrepreneurs’ risk preference will also increase, so as to achieve the purpose of improving enterprise innovation performance.

(4) Models 63–66 in Table 12 and 73–76 in Table 13 show that after adding VC’s innovation failure tolerance, the coefficient of VC’s shareholding ratio and dispatched directors decrease significantly. VC’s tolerance for innovation failure plays a partial mediating effect among its shareholding ratio, dispatched directors, and enterprise innovation performance. After controlling the sample selection effect, VC that holds corporate equity and sends directors still has a promoting effect on corporate innovation performance, which indicates that VC improves its tolerance for corporate technological innovation failure by holding corporate equity and sending directors to the board of directors after the event, and on this basis also improves corporate innovation performance.

### 6.2. Heckman two-step regression analysis

Since PSM can only control the effects of matched variables, there is no way to control other unobserved variables. Therefore, next, the Heckman two-step regression model will be used to control the influence of other unobserved variables. According to the studies of Xiong and Gui (2018), Guo and Jiang (2013), and other scholars, whether the enterprise is located in the Pearl River Delta, Yangtze River Delta and Beijing-Tianjin Region (Region) is used as the instrumental variable of VC participating enterprises in this paper.VC located in these regions developed relatively fast, and enterprises located in these areas are more likely to obtain VC funds. Therefore, in the first stage of the Heckman model, this variable are used as the instrumental variable for VC participation; Then, the unobtainable variable (Mills) that affects whether VC participates in the enterprise is obtained. Finally, in the second phase of the Heckman model, Mills is introduced as a control variable. The results are shown in Tables 14, 15.

**Table 14:** Heckman treatment effect of VC’s shareholding and dispatched directors on enterprise innovation performance.

Variable	Phase I	Phase II			
	VC Involvement	Patent		Innovation	
	Model 77	Model 78	Model 79	Model 80	Model 81
Region	0.159^***^ (0.038)				
VC Share		0.018^***^ (0.003)		0.085^***^ (0.002)	
Accredited Directors			0.239^***^ (0.030)		0.350^***^ (0.034)
Mills		0.323 (0.317)	0.209 (0.300)	0.128 (0.343)	−0.247 (0.318)
Stage		−0.074^***^ (0.024)	−0.076^***^ (0.023)	−0.140^***^ (0.027)	−0.095^***^ (0.024)
IT		−0.116^***^ (0.024)	−0.101^***^ (0.023)	−0.001 (0.027)	0.034 (0.025)
GDP Growth Rate		−0.015^**^ (0.006)	−0.010^*^ (0.006)	−0.013^**^ (0.007)	−0.007 (0.006)
Constant	0.459^***^ (0.031)	2.971^***^ (0.152)	3.030^***^ (0.145)	1.862^***^ (0.165)	2.326^***^ (0.155)
Year dummy	Control	Control	Control	Control	Control
Observations	5,119	3,202	3,644	3,158	3,644
Log likelihood	−3,063.5				
Log pseudolikelihood		−13,159.395	−14,930.628	−10,435.372	−12,592.09

The brackets are standard errors, and ***, **, and * are significant at the level of 0.01, 0.05, and 0.1, respectively.

**Table 15:** Heckman treatment effect of VC’s shareholding, dispatching directors and tolerance of failure on enterprise innovation performance (Phase II).

Variable	VC Tolerance	Patent		VC Tolerance		Innovation	Patent		Innovation	
	Model 82	Model 83	Model 84	Model 85	Model 86	Model 87	Model 88	Model 89	Model 90	Model 91
VC Share	0.105^***^ (0.014)			0.114^***^ (0.010)			0.004 (0.004)		0.080^***^ (0.003)	
Accredited Directors		0.432^***^ (0.149)			0.827^***^ (0.163)			0.197^***^ (0.040)		0.327^***^ (0.045)
VC Tolerance			0.046^***^ (0.007)			0.064^***^ (0.007)	0.059^***^ (0.008)	0.045^***^ (0.007)	0.035^***^ (0.007)	0.060^***^ (0.007)
Mills	−1.714 (1.654)	−1.997 (1.578)	0.849^**^ (0.411)	−0.312 (1.755)	−1.086 (1.696)	1.097^**^ (0.432)	1.115^**^ (0.443)	0.879^**^ (0.410)	1.013^**^ (0.455)	1.125^***^ (0.432)
Stage	1.264^***^ (0.126)	1.524^***^ (0.119)	−0.043 (0.032)	0.956^***^ (0.132)	1.181^***^ (0.128)	−0.096^***^ (0.032)	−0.056 (0.034)	−0.045 (0.032)	−0.126^***^ (0.034)	−0.094^***^ (0.032)
IT	0.049 (0.129)	0.103 (0.123)	−0.076^**^ (0.031)	0.058 (0.137)	0.200 (0.131)	0.071^**^ (0.034)	−0.092^***^ (0.034)	−0.073^**^ (0.031)	0.028 (0.036)	0.077^**^ (0.034)
GDP Growth Rate	0.032 (0.029)	0.05^*^ (0.028)	−0.005 (0.008)	0.029 (0.031)	0.050^*^ (0.030)	0.002 (0.008)	−0.006 (0.009)	−0.005 (0.008)	−0.008 (0.009)	−0.001 (0.008)
Constant	6.627^***^ (0.801)	6.823^***^ (0.764)	2.578^***^ (0.206)	5.655^***^ (0.849)	6.106^***^ (0.820)	1.709^***^ (0.216)	2.358^***^ (0.22)	2.532^***^ (0.205)	1.357^***^ (0.224)	1.652^***^ (0.215)
Year dummy	Control	Control	Control	Control	Control	Control	Control	Control	Control	Control
Observations	1,884	2,180	2,180	1,938	2,298	2,298	1,884	2,180	1,938	2,298
Adj *R*-squared	0.089	0.075		0.095	0.048					
*F*	37.76	36.28		41.64	23.99					
Log pseudolikelihood			−9,284.05			−8,321.048	−8,026.31	−9,273.553	−6,738.124	−8,297.463

The brackets are standard errors, and ***, **, and * are significant at the level of 0.01, 0.05, and 0.1, respectively.

In the first-stage model, the coefficient of Region is significantly positive at the level of 1%, indicating that if an enterprise is located in the Pearl River Delta, Yangtze River Delta, and Beijing and Tianjin, it is easier to obtain VC funds. In the second-stage regression model (for simplicity, only the results of the NBR regression model are listed, and the results of the multivariate regression model are similar to those of the NBR model), the results show that: (1) At the significance level of 1%, VC’s shareholding ratio and sending directors to the board of directors have a significant positive impact on the total number of patent applications and invention patent applications; (2) After VC’s tolerance for innovation failure is added, when the number of patent applications and the number of invention patent applications are taken as the explained variables, the coefficient of the VC’s shareholding ratio and dispatched directors both decrease (when the total number of patent applications is taken as the explained variable, the coefficient of shareholding ratio of VC is not significant). VC’s tolerance for innovation failure plays a mediating role in the relationship between its shareholding ratio, dispatched directors, and enterprise innovation performance.

Mills in model 78–81 are not significant, while Mills in model 88–91 are significant, indicating that there is a certain degree of self-selection problem, after controlling the self-selection bias, VC’s shareholding ratio and dispatched directors have significantly promoted enterprise innovation performance by tolerating enterprise innovation failure. Hypotheses H2-H3 still hold.

## 7. Conclusions and discussion

### 7.1. Research conclusion

Firstly, this paper study the influence of venture capital participation on enterprise innovation performance and the mechanism of venture capital impact on enterprise innovation performance from two aspects: shareholding ratio and dispatched directors to occupy the seats of enterprise board of directors. Secondly, we further study the mediating effect of venture capital institutions’ tolerance for innovation failure on the relationship between their shareholding, dispatched directors, and enterprise innovation performance. The main findings are as follows: (1) The participation of venture capital will have a significantly positive impact on the innovation performance of enterprises. It promotes the innovation performance of enterprises by holding corporate equity and sending directors to the board of directors. The higher the shareholding ratio of venture capital is, the directors dispatched to the board of directors will effectively supervise and control the enterprise, give technical guidance in the process of enterprise innovation, rationally allocate various resources used in the process of enterprise innovation, and ultimately improve the performance of enterprise innovation. (2) Venture capital’s tolerance for innovation failure plays a partial mediating role in the relationship between its shareholding ratio, dispatched directors, and enterprise innovation performance. By holding shares of enterprises and sending directors to occupy seats on the board of directors, venture capital improves their tolerance for the failure of technological innovation of enterprises, urges enterprises to optimize various processes of project innovation, finally, it helps enterprises achieve the purpose of improving innovation performance. (3) As joint investment can reduce the investment risks faced by venture capital, their tolerance for innovation failure will also increase, and the funds they provide to enterprises will also increase. Accordingly, enterprises will also carry out more R&D on high-risk technologies and products, which is ultimately conducive to improving the innovation performance of enterprises. (4) In the case of close-range investment, with the increase of the tolerance of venture capital to enterprise innovation failure, entrepreneurs’ preference for risk will also increase, which will help them better support enterprise innovation.

### 7.2. Countermeasures and suggestions

This paper also puts forward some suggestions from the perspective of practice: (1) By holding shares of enterprises and sending directors to occupy board seats, venture capital institutions improve their tolerance for the failure of early technological innovation, and ultimately promote enterprise innovation. Therefore, venture capital should be encouraged to actively participate in corporate governance. By increasing the proportion of shareholding and dispatching directors, venture capital should improve its tolerance of innovation failure, which can ultimately improve the efficiency of enterprise innovation. (2) Venture capital institutions with different knowledge, experience, and relationship networks, as well as with different geographical preferences, can be encouraged to join together to invest in companies. Strengthening the communication among investment institutions, so that they can provide complementary value-added services to enterprises to improve the innovation ability and performance of enterprises. (3) The government should improve the laws and regulations on intellectual property protection and other related aspects, create a good legal environment, help innovative enterprises to innovate more actively.

### 7.3. Limitations and future research direction

This paper also has certain shortcomings: due to the problem of data availability, this paper mainly adopts the data of patent applications when measuring the innovation performance of enterprises, which will have a certain influence on the research conclusion. In view of this deficiency, follow-up research can expand the scope of data collection, further obtain data on enterprise innovation investment, patent authorization and other aspects, and comprehensively measure the performance of enterprise innovation, which can better improve the reliability of research conclusions.

### 7.4. Theoretical contributions

Firstly, although some scholars have focused on the impact of venture capital participation on firms’ innovation, they have not conducted further in-depth studies on the mechanisms by which venture capital affects firms’ innovation performance. This paper is based on innovation economics and organizational control theory, the impact of venture capital participation on enterprise innovation performance is studied, and the mechanism of venture capital on enterprise innovation performance is studied from two aspects: shareholding ratio and sending directors to occupy seats on the corporate board of directors. Secondly, although some scholars have paid attention to the fact that the attitude of venture capital to innovation failures of enterprises may affect the risk appetite of entrepreneurs, they have not further studied the influence of venture capital’ tolerance for innovation failures of enterprises on the relationship between venture capital and enterprise innovation performance. This article is based on the perspective of enterprise innovation culture, the mediating effects between venture capital firms’ tolerance of innovation failure in terms of shareholding, the appointment of directors to occupy board seats, and firms’ innovation performance are further investigated. Thirdly, some studies have paid attention to the impact of the type, investment strategy of venture capital institutions on the innovation performance of enterprises, some studies have ignored that different types of venture capital have different characteristics due to their own characteristics, and their tolerance for innovation failure of enterprises and the innovation performance of enterprises will have differentiated moderating effects, from the perspective of heterogeneity of different types of investment institutions, the moderating effect of characteristics such as joint investment strategies and geographical proximity of investment institutions on the relationship between failure tolerance of venture capital and firm innovation performance is investigated. Fourthly, to better identify the “selection effect” and “value-added effect” of venture capital particiption, this paper also adopts the PSM method and Heckman treatment effect model to further test the robustness of the impact of the VC’s shareholding ratio, dispatched directors on the innovation performance of enterprises, and the mediating effect of failure tolerance of venture capital institutions. Finally, Based on relevant psychological theories, this paper studies the impact of venture capital’ psychological capital on innovation performance by influencing their tolerance for innovation failure. It provides a theoretical basis for in-depth study of the relationship between the participation of venture capital, the tolerance of venture capital for the innovation failure of enterprises and the innovation performance of enterprises, and also provides experience reference for the establishment of enterprise culture in fault tolerance and the promotion of enterprise scientific and technological innovation.

## Data availability statement

The original contributions presented in the study are included in the article/Supplementary material, further inquiries can be directed to the corresponding author.

## Author contributions

JH was responsible for writing—original draft, formal analysis, methodology, and conceptualization of this study. MC contributed to paper writing and hypothesis model design. YC Participated in the paper translating. QJ participated in the paper writing and analyzed data. SW collected and screened literature. All authors contributed to the article and approved the submitted version.

## Funding

This study was supported by the following fund projects, namely Humanities and Social Sciences Research Youth Foundation of Ministry of Education of China (18YJC630046), Shaanxi Soft Science Research Program (2020KRM132).

## Conflict of interest

The authors declare that the research was conducted in the absence of any commercial or financial relationships that could be construed as a potential conflict of interest.

## Publisher’s note

All claims expressed in this article are solely those of the authors and do not necessarily represent those of their affiliated organizations, or those of the publisher, the editors and the reviewers. Any product that may be evaluated in this article, or claim that may be made by its manufacturer, is not guaranteed or endorsed by the publisher.
