# Supplementary material for: The study on the relationship between venture capital, tolerance to failure, and enterprise innovation performance
Source: Front Psychol. 2023 Feb 15;14:1133324. doi: 10.3389/fpsyg.2023.1133324 (PMC9975738; doi:10.3389/fpsyg.2023.1133324)
Supplement: Supplementary file 1 [file Data_Sheet_1.pdf]

Supplementary Figures and Tables

1.Figures

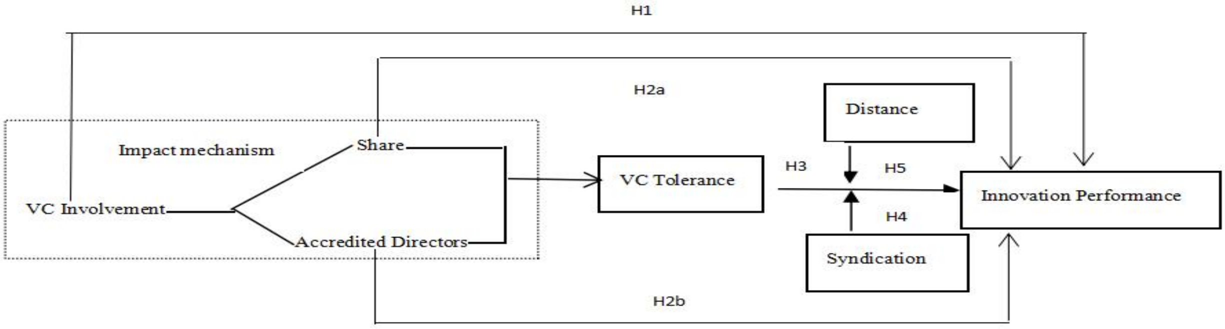

Figure 1 Theoretical Model

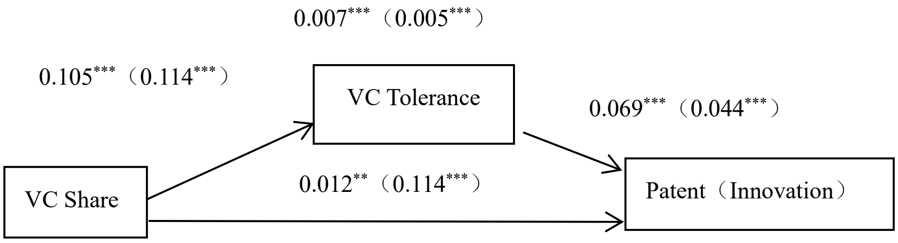

Figure 2 The intermediary effect of VC's tolerance for innovation failure between VC shareholding ratio and enterprise patent application

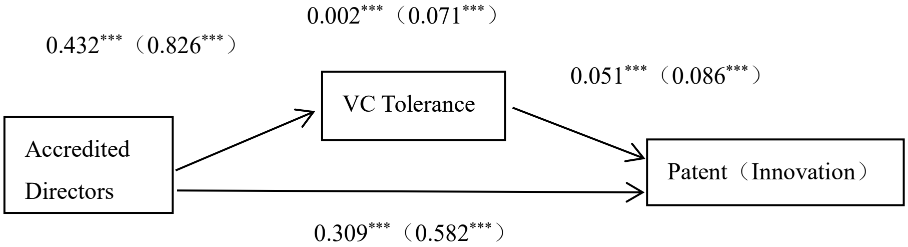

Figure 3 The intermediary effect of VC's tolerance for innovation failure between VC dispatched directors and enterprise patent applications

2.Tables

Table 1 Descriptive statistics of main variables

	Observations	Minimum value	Maximum	Mean value	Standard Deviation
VC Involvement	5119	0	1	0.712	0.453
Patent	5119	2	102	20.289	27.187
Innovation	5119	0	36	7.052	10.061
VC Share	4520	0	15.53	3.870	4.253
Accredited Directors	5119	0	1	0.169	0.375
VC Tolerance	2848	1.518	12	6.503	3.042
Syndication	5119	1	12	4.676	3.109
Distance	4454	0	8.033	5.205	3.029
Stage	5119	0	1	0.4500	0.5
IT	5119	0	1	0.388	0.487
GDP	5119	-5	3.7	1.663	2.096

Table 2 Pearson correlation analysis of the main variables

	VC Share	Accredited Directors	Patent	Innovation	VC Tolerance	Stage	IT	GDP
VC Share	1	0.356**	0.469**	0.575**	0.268**	0.057**	0.039*	-0.028
Accredited Directors	0.356**	1	0.136**	0.180**	0.104**	0.015	0.006	-0.031
Patent	0.469**	0.136**	1	0.867**	0.217**	-0.061**	-0.076**	-0.033*
Innovation	0.575**	0.180**	0.867**	1	0.197**	-0.066**	0.017	-0.027
VC Tolerance	0.268**	0.104**	0.217**	0.197**	1	0.192**	0.044*	0.033
Stage	0.057**	0.015	-0.061**	-0.066**	0.192**	1	0.063**	-0.002
IT	0.039*	0.006	-0.076**	0.017	0.044*	0.063**	1	-0.049**
GDP	-0.028	-0.031	-0.033*	-0.027	0.033	-0.002	-0.049**	1

Note: **、*They are significant at the level of 0.01 and 0.05 respectivel
